# Phase Behavior of a Carbon Dioxide/Methyl Trimethoxy Silane/Polystyrene Ternary System

**DOI:** 10.3390/polym11020246

**Published:** 2019-02-02

**Authors:** Hiroaki Matsukawa, Satoshi Yoda, Yasuo Okawa, Katsuto Otake

**Affiliations:** 1 Department of Industrial Chemistry, Faculty of Engineering, Tokyo University of Science, Ichigaya Funakawara-machi 12-1, Shinjuku-ku, Tokyo 162-0826, Japan; hmatsukawa@ci.kagu.tus.ac.jp; 2 Nanosystem Research Institute, Research Institute for Chemical Process Technology, National Institute of Advanced Industrial Science and Technology, Tsukuba Central 5, Higashi 1-1-1, Tsukuba, Ibaraki 305-8565, Japan; s-yoda@aist.go.jp; 3 Showa Jyushi Co. Ltd., Sekishinden, Yoshikawa, Saitama 342-0014, Japan; y.okawa@shouwa-jp.com

**Keywords:** vapor-liquid equilibrium, liquid-liquid equilibrium, vapor-liquid-liquid equilibrium, carbon dioxide, methyl trimethoxy silane, polystyrene, synthetic method

## Abstract

Recently, polymeric foams filled with a silica aerogel have been developed. The phase behavior of CO_2_/silicon alkoxide binary systems and CO_2_/silicon alkoxide/polymer ternary systems is an important factor that affects the design of novel processes. The phase behavior of a carbon dioxide (CO_2_)/methyl trimethoxy silane (MTMS)/polystyrene (PS) ternary system was measured using a synthetic method involving the observation of the bubble and cloud point. The phase boundaries were measured at temperatures ranging from 313.2 to 393.2 K and CO_2_ weight fractions between 0.01 and 0.08. The CO_2_/MTMS/PS system showed a similar CO_2_ mass fraction dependence of the phase behavior to that observed for the CO_2_/tetramethyl orthosilicate (TMOS)/PS system. When the phase boundaries of these systems were compared, the vapor-liquid (VL) and vapor-liquid-liquid (VLL) lines were found to be nearly identical, while the liquid-liquid (LL) lines were different. These results indicate that the affinity between the silicon alkoxide and polymer greatly influences the liquid-liquid phase separation.

## 1. Introduction

An aerogel is a porous material prepared by replacing the solvent within a gel with a gas upon supercritical drying without destroying its structure [1]. The first example of aerogel was reported by Kistler and co-workers in 1931 [2], and consisted of silica, alumina, nickel tartrate, tungsten oxide, gelatin, agar, nitrocellulose, cellulose, and ovalbumin.

Aerogels with a porosity of more than 90% are promising materials due to their exceptional lightweightness and thermal insulation properties. Especially, while the thermal conductivity of a general insulator is about 20 to 45 mW/(m K), the thermal insulation property of aerogels was reported to be lower [3]. In particular, their thermal conductivity is the lowest among free-standing solids due to their fine porous structure. The mean free path of nitrogen molecules at room temperature is about 70 nm. If pores smaller than this are formed, the convection of gases as well as the molecular thermal momentum exchange does not occur within an aerogel. In other words, an aerogel possesses a thermal insulation close to that of vacuum. Therefore, it is considered for use as thermal insulator in industrial applications. In recent years, it has been used for the preparation of thermal insulating glass. It is prepared by dispersing the aerogel particles in glass or by sandwiching and pressure bonding block-shaped aerogel with glass [4,5].

In the 1990s, the National Aeronautics and Space Administration (NASA) succeeded in the practical use of aerogels. However, the utilization of aerogels has not been widespread so far, most likely as a consequence of their preparation process, which requires specialized equipment such as a supercritical dryer and high costs. Kanamori et al. reported a novel and more cost-effective aerogel preparation method that avoids the use of supercritical drying [6]. In particular, they developed high-strength aerogels based on a poly(methyl silsesquioxane) (PMSQ, CH_3_SiO_1.5_) network obtained from methyltri(methoxy)silane (MTMS). Such material exhibited a transparency (transmission of more than 90% of visible light through 10 mm thickness) and thermal insulation property (thermal conductivity of about 14 mW/(m K)) equivalent to that of silica aerogels [7,8].

Another factor that hampers the general applicability of aerogels is their brittleness, which is due to their porous structure. Such brittleness is the reason why supercritical drying is required rather than ordinary drying for the production of aerogels. However, if the skeleton was made thicker to increase the strength, the advantages of the aerogels would be lost. Therefore, hybridization with fibers and polymers was proposed as solution to reinforce the aerogel skeleton [9,10,11]. However, a consequential disadvantage is that the thermal conductivity increases, or the aerogel becomes not transparent.

Another method to protect aerogels from impact consists of using a thermal insulating glass as described above. Polymeric foams are also one of the potential materials that can be used as thermal insulators [12,13,14,15]. However, at present the thermal insulation performance of single polymeric foams is insufficient. Recently, polymeric foams filled with a silica aerogel have been developed [16]. This approach is expected to reinforce the brittleness of the silica aerogel, while compensating for the low thermal insulation property of the polymeric foam.

It is difficult to knead a silica aerogel within a polymer due to its brittleness. Therefore, silica aerogel-polymeric foam composite materials need to be continuously produced, thus leading to a drastic cost reduction. The mass production of these composite materials can be achieved by carbon dioxide (CO_2_)-assisted extrusion of a polymer and silicon alkoxide, which is a raw material used in the production of silica aerogels. At the exit of the extruder, nucleation and growth of CO_2_ bubbles occur from the thermodynamically stable ternary mixture of CO_2_/silicon alkoxide/polymer as the pressure decreases. The bubbles thus formed are gradually filled with a silicon alkoxide-rich liquid that bleeds from the bubble walls, which is gelated by subsequent hydrolysis in humid conditions and/or by a catalyst in air.

The structure of a composite material is mainly determined by the initial separation of CO_2_ from the mixture, which depends on the phase behavior of the system. Therefore, the phase behavior of CO_2_/silicon alkoxide binary systems and CO_2_/silicon alkoxide/polymer ternary systems is an important factor that affects the design of novel processes. Previously, data about CO_2_/tetramethoxysilane (TMOS)/polymethyl methacrylate (PMMA) or polystyrene (PS) ternary systems have been reported [17]. It was found that the phase behavior of these CO_2_/silicon alkoxide/polymer ternary systems changed with different polymer species. The combination of a silicon alkoxide and polymer is closely related to the function of the final composite material. It is also necessary to pay attention to the influence of the silicon alkoxide species on the phase behavior and the corresponding data are needed. The phase behavior of CO_2_/TMOS [18], CO_2_/MTMS, and CO_2_/methyl silicate 51 (MS-51) systems [19] have already been reported. In this work, the phase behavior of a CO_2_/MTMS/PS ternary system was measured over a wide range of temperatures, pressures, and polymer mass fractions using a synthetic method. The effects of molecular weight of the polymer, polymer mass ratio to MTMS, and silicon alkoxide species on the phase behavior were also examined. The synthetic method is one of the methods to grasp the phase behavior which is suitable for complex systems containing CO_2_ because it can visually observe the phase state. Actually, Santos et al. measured the phase behavior of a CO_2_/methyl methacrylate (MMA)/polydimethylsiloxane (PDMS) system using a synthetic method [20]. Furthermore, this method has been used to deduce the phase diagram of complex systems containing CO_2_ [21,22,23,24].

## 2. Materials and Methods

### 2.1. Materials

Carbon dioxide (CO_2_, CAS number [124-38-9], purity > 99.99%) was purchased from Showa Yozai Co. (Osaka, Japan), methyltri(methoxy)silane (MTMS, CAS number [1185-55-3], purity > 98.0%) was acquired from Tokyo Kasei Co. (Tokyo, Japan), while polystyrenes (PS, CAS number [9003-53-6]) with different molecular weights were obtained from Sigma Aldrich Co. (M_W_ = 35,000, M_W_/Mn = 2.020, St. Louis, MO, USA) and Kanto Kagaku Co. (M_W_ = 250,000, M_W_/Mn = 2.348, Tokyo, Japan). All the materials employed in this study are listed in Table 1 and were used as received.

### 2.2. Experimental Apparatus and Procedure

In this study, the ternary system phase diagram was reduced using a synthetic method. The details of the experimental apparatus and procedures employed have been described elsewhere [18].

In brief, appropriate amounts of PS, MTMS, and CO_2_ were introduced into the front part of a variable-volume view cell containing a moving piston. The composition in the front part of the cell was calculated from the mass of the materials. The cell was heated to the desired temperature and then pressurized by moving the piston from the back part of the cell until a single phase was achieved. Next, the pressure was slowly decreased until the phase separation occurred. The phase separation points were visually observed through the view window. In this system, the cloud-point (CP) and bubble point (BP) were confirmed as the phase separation points. The CP is defined as the point at which the solution becomes so opaque that it is no longer possible to see the magnetic stirring tip in the cell. The BP is defined as the point at which small bubbles appear in the cell. Upon returning to the state before pressurization, the mixture was re-pressurized to obtain the single phase again. This process was repeated at least five times, and the numerical average of the values excluding the maximum and minimum values was determined.

For all the measurements, the total uncertainties were ±0.1 K, ±0.01 MPa, and ±1.32 × 10^−3^ (CO_2_/MTMS/PS system) in terms of temperature, pressure, and composition, respectively. The details of the uncertainties have been previously described [17], and the calculated composition errors are listed in Table 2, Table 3, Table 4 and Table 5.

## 3. Results and Discussion

**Figure 1:**
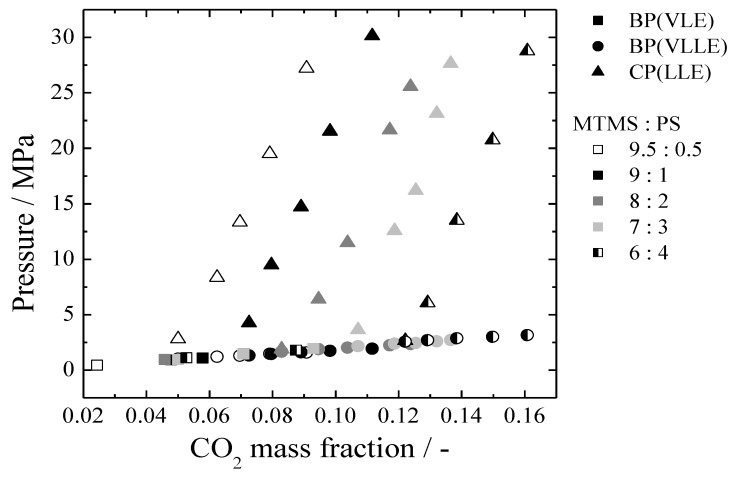
Phase behavior of a CO_2_/MTMS/PS ternary system (M_W_ = 35,000, 313.2 K).

**Figure 2:**
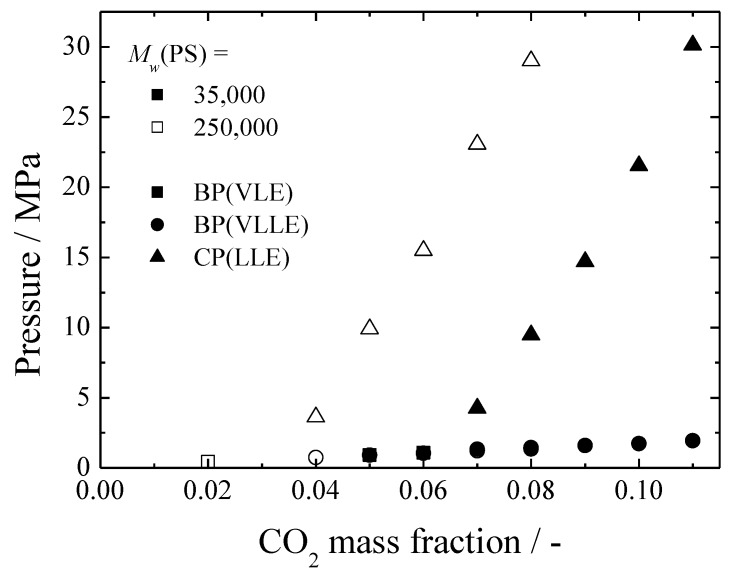
Effect of the polymer molecular weight on the phase behavior of a CO_2_/MTMS/PS ternary system (313.2 K, MTMS:PS = 9:1).

**Figure 3:**
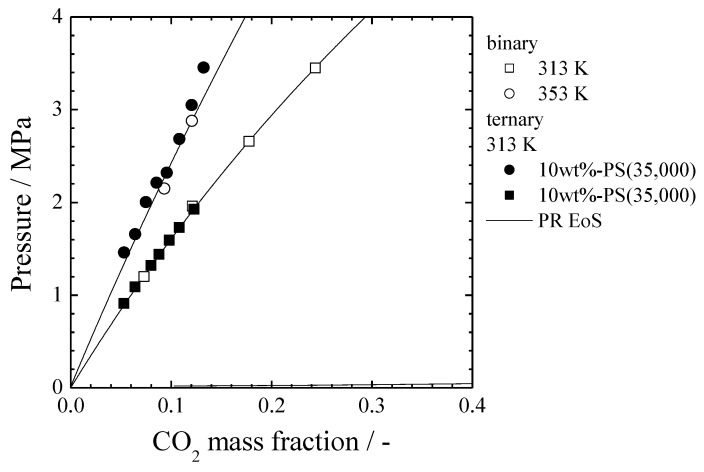
Comparison between CO_2_/MTMS/PS (M_W_ = 35,000) ternary and CO_2_/MTMS binary systems.

**Figure 4:**
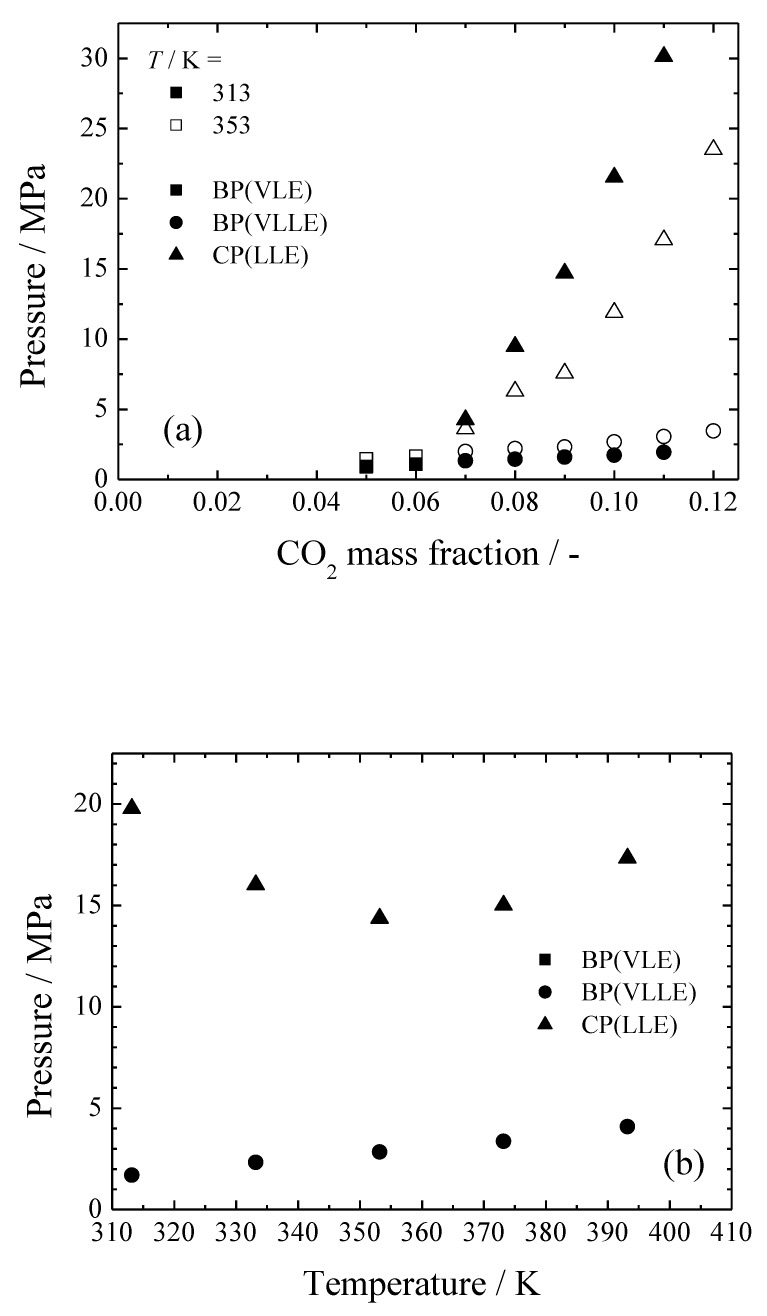
Effect of the temperature on the phase behavior of a CO_2_/MTMS/PS ternary system (M_W_ = 35,000, MTMS:PS = 9:1). (**a**) Pressure-CO_2_ mass fraction phase diagram. (**b**) Pressure-temperature phase diagram (*m*_1_ = 0.094, *m*_2_ = 0.814, *m*_3_ = 0.091).

**Figure 5:**
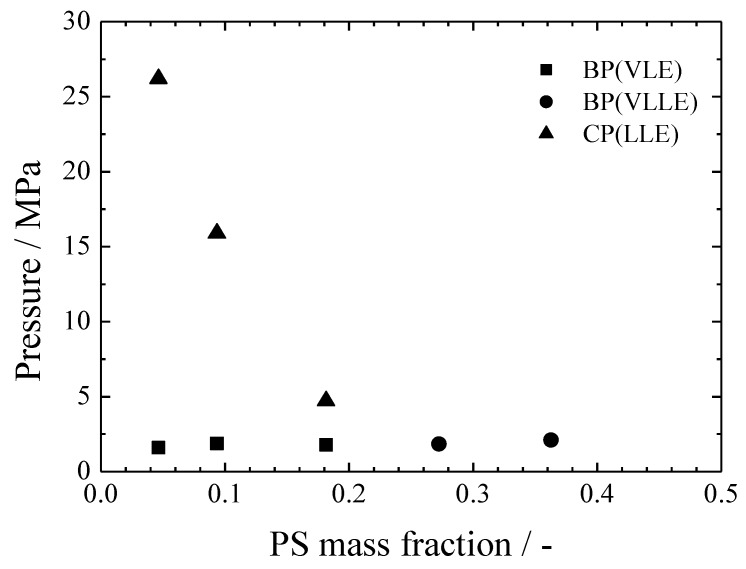
Effect of the MTMS:PS ratio on a CO_2_/MTMS/PS ternary system at 313.2 K and constant CO_2_ mass fraction of 0.09.

**Figure 6:**
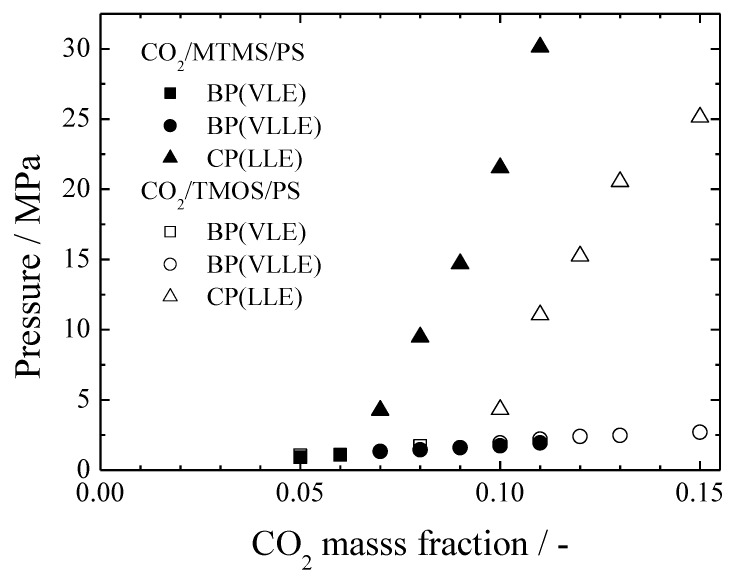
Comparison between CO_2_/MTMS/PS and CO_2_/TMOS/PS ternary systems (313.2 K, M_W_ = 35,000, silicon alkoxide:PS = 9:1).

The experimental results are shown in Figure 1, Figure 2, Figure 3, Figure 4, Figure 5 and Figure 6 and summarized in Table 2, Table 3, Table 4 and Table 5. Figure 1 shows a typical phase diagram of a CO_2_/MTMS/PS ternary system. The horizontal axis represents the CO_2_ mass fraction, while the vertical axis indicates the pressure. The CO_2_/MTMS/PS system exhibited two different types of phase behavior with changes in the CO_2_ mass fraction. Such behavior is similar to that of CO_2_/TMOS/polymer systems, whose details have been previously described [17]. Furthermore, this behavior is similar to that of CO_2_/MMA/PDMS systems also reported by Santos et al. [20].

### 3.1. Effects of Experimental Parameters

The effects of the molecular weight, temperature, and PS ratio to MTMS on the phase behavior of the CO_2_/MTMS/PS ternary system was found to be similar to that observed for a CO_2_/TMOS/PS ternary system [17]. The main features of the CO_2_/MTMS/PS ternary system are discussed below.

Figure 2 shows the effect of the polymer molecular weight on the CO_2_/MTMS/PS system. With increases of the polymer molecular weight, the vapor-liquid (VL) and the vapor-liquid-liquid (VLL) lines did not change, while the liquid-liquid (LL) line shifted towards the lower CO_2_ mass fraction. The VL lines of a CO_2_/MTMS binary system [19] as well as the VL and VLL lines of the CO_2_/MTMS/PS ternary system were compared using polymer-free bases. As shown in Figure 3, the solid lines indicate the vapor-liquid equilibrium of the binary system calculated using the Peng-Robinson equation of state (PR EoS). The details of this calculation are described in a previous report [19]. Moreover, wt%-polymer denotes the mass fraction of the polymer in MTMS. As it can be seen, the VL lines of the binary system are almost identical to the VL and VLL lines of the CO_2_/MTMS/PS system. Based on this comparison, it could be deduced that the polymer was not involved in the separation of the gas phase from the uniform mixture. Therefore, the VL and VLL lines in Figure 2 did not change even if the polymer molecular weight changed. On the other hand, when the polymer molecular weight varied, the LLE line also changed, indicating that the polymer greatly influenced the LL separation.

Figure 4 shows the effect of the temperature on the CO_2_/MTMS/PS ternary system. As seen in Figure 4a, upon increasing of the temperature, the VL and VLL lines shifted to higher pressures, analogous to the behavior of the CO_2_/MTMS binary system [19]. On the other hand, the LL line shifted to lower pressures. Such behavior is similar to that of the CO_2_/TMOS/PS ternary system, while being opposite to that of the CO_2_/TMOS/PMMA ternary system [17]. This might be due to a marked increase of the solubility of the polymer in the case of MTMS. It could be seen that the LL separation pressure was greatly influenced by the affinity between the three components.

Figure 4b shows the pressure-temperature phase diagram of the CO_2_/MTMS/PS ternary system. In general, the phase diagrams of supercritical fluid/polymer systems are reported as pressure-temperature phase diagrams [25]. Phase diagrams are classified according to the behavior of the cloud point (CP, LL phase separation point) relative to the temperature. As shown in Figure 4b, the CP initially decreased with the increase of the temperature, and later increased. This is a U-LCST-type behavior, which takes place when the upper critical solution temperature (UCST)-type and the lower critical solution temperature (LCST)-type behaviors occur simultaneously. The phase behavior of the polymer solutions depends on the energy interactions and free volume differences between the polymer and the solvent. The concentration of the co-solvent has a great effect on the phase behavior type. Furthermore, the phase behavior is expected to change with the proportion of each component. Actually, Lee et al. reported that the phase behavior changes from the UCST-type to the LCST-type as co-solvent concentration increased in the CO_2_/dodecyl acrylate/polydodecyl acrylate systems [24].

Figure 5 shows the effect of the MTMS:PS weight ratio on the phase behavior of the CO_2_/MTMS/PS ternary system at 313.2 K with a constant CO_2_ mass fraction of 0.09 for the mixture. Each point was interpolated from Table 2 and Figure 1. With the increase in the polymer mass fraction, the CP decreased, and the homogeneous phase region was enlarged. This tendency is similar to that of the CO_2_/TMOS/PS system, but opposes that of the CO_2_/TMOS/PMMA system [17]. The LL phase separation greatly depends on the relationships between the three components, as mentioned above. Sometimes, depending on the component species, an opposite tendency can be observed. In this study, it seemed that the effect of the polymer species was significant, although the specific cause remains unknown. Further investigations to elucidate the causes of these phenomena are underway in our laboratory.

### 3.2. Comparison with the CO_2_/TMOS/PS System

Figure 6 shows the difference of the phase behavior depending on the silicon alkoxide species. The comparison between ternary and binary systems revealed that the polymer was not involved in the gas phase separation. Furthermore, it was found in previous studies that the VL lines of CO_2_/TMOS and CO_2_/MTMS binary systems almost coincided [19]. In other words, the removal of an oxygen atom from one of the methoxy groups of TMOS had no effect on the phase diagram, and no difference in affinity was observed between CO_2_ and TMOS or MTMS. As a result, the gas phase separation (VL and VLL) lines of the system including TMOS almost agreed with those of MTMS.

On the other hand, the effect of the silicon alkoxide species could be seen in the LL lines. The LL line of MTMS was separated from the VL line at a lower CO_2_ mass fraction compared to TMOS, and the homogeneous phase of MTMS was smaller. Hereafter, the affinity between each component was considered. Since CO_2_-PS was common in both systems, and the affinity between CO_2_ and silicon alkoxide was almost the same from the results of the VL lines; only the affinity between the silicon alkoxide and PS could affect the LL line. As described above, when the affinity was lowered and the polymer molecular weight decreased, the homogeneous phase region narrowed. As a consequence, it could be assumed that TMOS had a better affinity to PS than MTMS.

The affinity between the components can be discussed in view of the solubility parameter [26,27]. There are one to three component solubility parameters. In this case, the one component solubility parameter (Hildebrand parameter) [28,29] was adopted. Unfortunately, the solubility parameters of the silicon alkoxide were not reported. Therefore, they were calculated by the group contribution method reported by Fedors [30]. The solubility parameters in this work [27,30,31] are listed in Table 6. The smaller the solubility parameter difference is, the better the affinity between the components. As shown in Figure 5, TMOS is closer to PS than MTMS. Thus, TMOS had a better affinity to PS than MTMS, which is consistent with the results of the phase behavior.

The solubility parameter of the components increased in the order of silicon alkoxide < polymer < CO_2_, and the relative relationship did not change depending on the silicon alkoxide and polymer species. Unfortunately, it is difficult to explain the effect of the polymer species according to the one component solubility parameter.

## 4. Conclusions

A phase diagram for a CO_2_/MTMS/PS ternary system was obtained over a wide range of temperatures, pressures, and polymer mass fractions. The effects of the polymer molecular weight, temperature, and MTMS:PS ratio showed a similar tendency as that observed for the CO_2_/TMOS/PS ternary system. However, the LL line of MTMS separated from the VL line at a lower CO_2_ mass fraction in contrast to the case of TMOS. This could be explained from the difference in affinity between the silicon alkoxide and PS, which also agreed with the trend found for the solubility parameter. The phase behavior varied with the silicon alkoxide species, and the preparation conditions of the polymeric foam changed. This information is important for the design and development of novel processes. Unfortunately, it is difficult to explain the effect of the polymer species according to single component solubility parameters. Further investigations to elucidate the causes of these phenomena are currently underway in our laboratory.

## Figures and Tables

**Table 1:** Specifications of the pure components.

**Component**	**CAS Number**	**Supplier**	**Mole Fraction Purity (Supplier)**	
Carbon dioxide, CO_2_	124-38-9	Showa Yozai Co.	0.9999	
Methyltri(methoxy)silane, MTMS	1185-55-3	Tokyo Kasei Co.	0.98	
**Component**	**CAS Number**	**Supplier**	**M_W_**	**M_W_/Mn**
Polystyrene, PS	9003-53-6	Sigma Aldrich Co.	35,000	2.020
		Kanto Kagaku Co.	250,000	2.348

**Table 2:** Experimental data for a CO_2_(1)/MTMS(2)/PS(3) system (M_W_ = 35,000, 313.2 K).

*m*_1_ (CO_2_) /-	*m*_2_ (MTMS) /-	*m*_3_ (PS) /-	BP (VLE) /MPa	BP (VLLE) /MPa	CP (LLE) /MPa	±Δ*m*_1_ × 10^3^ /-
MTMS:PS = 9.5:0.5						
0.024	0.926	0.050	0.43			1.31
0.050	0.902	0.048		1.02	2.79	1.26
0.062	0.890	0.048		1.20	8.36	1.24
0.070	0.883	0.047		1.30	13.34	1.24
0.079	0.874	0.047		1.46	19.51	1.23
0.091	0.863	0.046		1.59	27.22	1.22
MTMS:PS = 9:1						
0.048	0.854	0.098	0.91			1.26
0.058	0.845	0.097	1.09			1.25
0.073	0.832	0.095		1.32	4.26	1.23
0.080	0.826	0.095		1.44	9.47	1.23
0.089	0.817	0.094		1.59	14.70	1.22
0.098	0.809	0.093		1.73	21.53	1.22
0.112	0.797	0.091		1.93	30.13	1.22
MTMS:PS = 8:2						
0.046	0.764	0.190	0.95			1.27
0.071	0.744	0.185	1.46			1.24
0.083	0.734	0.183		1.65	1.88	1.23
0.095	0.725	0.181		1.89	6.38	1.22
0.104	0.717	0.179		2.01	11.48	1.22
0.117	0.707	0.176		2.24	21.64	1.22
0.124	0.701	0.175		2.35	25.56	1.22
MTMS:PS = 7:3						
0.050	0.665	0.284	0.95			1.26
0.071	0.651	0.278	1.44			1.24
0.093	0.635	0.272	1.95			1.22
0.107	0.625	0.267		2.15	3.63	1.22
0.119	0.617	0.264		2.39	12.56	1.22
0.125	0.613	0.262		2.44	16.20	1.22
0.132	0.608	0.260		2.60	23.13	1.22
0.136	0.605	0.259		2.70	27.63	1.22

**Table 3:** Experimental data for a CO_2_(1)/MTMS(2)/PS(3) system (M_W_ = 35,000, 353.2 K).

*m*_1_ (CO_2_) /-	*m*_2_ (MTMS) /-	*m*_3_ (PS) /-	BP (VLE) /MPa	BP (VLLE) /MPa	CP (LLE) /MPa	±Δ*m*_1_ × 10^3^ /-
MTMS:PS = 9:1						
0.048	0.856	0.096	1.46			1.26
0.058	0.847	0.095	1.66			1.25
0.068	0.838	0.094		2.00	3.60	1.24
0.077	0.829	0.093		2.21	6.28	1.23
0.087	0.821	0.092		2.32	7.58	1.22
0.098	0.810	0.091		2.68	11.91	1.22
0.110	0.800	0.090		3.05	17.07	1.22
0.121	0.790	0.089		3.45	23.51	1.22

**Table 4:** Experimental data for a CO_2_(1)/MTMS(2)/PS(3) system (M_W_ = 250,000, 313.2 K).

*m*_1_ (CO_2_) /-	*m*_2_ (MTMS) /-	*m*_3_ (PS) /-	BP (VLE) /MPa	BP (VLLE) /MPa	CP (LLE) /MPa	±Δ*m*_1_ × 10^3^ /-
MTMS:PS = 9:1						
0.020	0.881	0.099	0.43			1.32
0.039	0.864	0.097		0.74	3.62	1.28
0.049	0.855	0.096		0.92	9.91	1.26
0.062	0.843	0.095		1.05	15.50	1.25
0.072	0.834	0.094		1.22	23.06	1.24
0.082	0.826	0.093		1.34	29.01	1.23
MTMS:PS = 8:2						
0.032	0.774	0.194	0.70			1.29
0.057	0.754	0.189		1.08	6.60	1.25
0.066	0.747	0.187		1.28	13.83	1.24
0.075	0.740	0.185		1.41	18.01	1.23
0.080	0.736	0.184		1.60	24.10	1.23
0.091	0.727	0.182		1.70	30.67	1.22

**Table 5:** Experimental data for a CO_2_(1)/MTMS(2)/PS(3) system (M_W_ = 35,000, *m*_1_ = 0.094, *m*_2_ = 0.815, *m*_3_ = 0.091, ±Δ*m*_1_ × 10^3^ = 1.22).

*T*/K	BP (VLLE) /MPa	CP(LLE) /MPa
313.2	1.70	19.78
333.2	2.32	16.02
353.2	2.84	14.36
373.2	3.36	15.01
393.2	4.09	17.34

**Table 6:** Hildebrand solubility parameters of the pure components.

Component	δ/MPa^1/2^
CO_2_	4.70 ^(1)^
TMOS	15.4 ^(2)^
MTMS	14.9 ^(2)^
PS	17.4~19.0 ^(3)^
PMMA	19.9~21.2 ^(3)^

^(1)^ 313.2 K, 7 MPa, reported by Marcus [27]. ^(2)^ Estimated as mentioned in the text [26]. ^(3)^ Reported by Krevelen [23].
